# The Pick-and-Roll in Basketball From Deep Interviews of Elite Coaches: A Mixed Method Approach From Polar Coordinate Analysis

**DOI:** 10.3389/fpsyg.2022.801100

**Published:** 2022-03-28

**Authors:** Hermilo Nunes, Xavier Iglesias, Luca Del Giacco, M. Teresa Anguera

**Affiliations:** ^1^National Institute of Physical Education of Catalonia, University of Barcelona, Barcelona, Spain; ^2^Department of Social Psychology and Quantitative Psychology, University of Barcelona, Barcelona, Spain; ^3^Faculty of Psychology, Institute of Neurosciences, University of Barcelona, Barcelona, Spain

**Keywords:** ball screen, tactics, basketball, coaches, systematic observation, pick and roll, offensive and defensive, mixed methods approach

## Abstract

Pick-and-roll is the most widespread cooperative action among high-level basketball teams and the most applied strategy by coaches to gain an advantage over the rival team. During pick-and-roll, opposing teams perform antagonistic actions based on goals that are expressed in offensive and defensive tactics. The aim of this study is to examine the approaches of high-level coaches on the offensive and defensive dynamics emerging in matches of a basketball elite team during an entire season of the Spanish *Asociación de Clubes de Baloncesto* (ACB) league. To this end, we used a mixed-methods approach based on systematic observation of verbatim transcripts of interviews conducted with six high-level coaches about the pick-and-roll dynamics that emerged in matches of the Unicaja Málaga team during an entire season of the ACB league. The observational design was nomothetic, punctual, and multidimensional. The choice of this methodology is justified since we developed an *ad hoc* indirect observation tool to evaluate the coaches’ perspective on this dynamic. Once the intra-observer reliability of the instrument was confirmed, we performed a polar coordinate analysis to identify the significant relationships between the coaches’ evaluations and the offensive and defensive pick-and-roll elements that supported such verbal behaviors. The results highlight the presence of various offensive and defensive aspects of pick-and-roll (*n* = 2224) emerging in the Unicaja team that were significantly associated with positive and negative evaluations of the coaches. The interview confirms that coach 1 and his staff were less confident in options that pick-and-roll offer, which is also reflected in the record of screens made and simulated, than coach 3. This study shows that the application of mixed methods, by analysis of the polar coordinate of the coding carried out on responses of a systematized interview, has proven to be an effective strategy in obtaining relevant information on the expert knowledge of the elite coaches on the influence of pick-and-roll on tactical actions in basketball.

## Introduction

During basketball matches in a high-level league, teams have antagonistic goals that are conditioned by whether or not they have the ball, resulting in the attack and defense phases (Bayer, 1992). These phases involve coordinated actions resulting from differentiated mental processes that are performed in technically and tactically elaborated procedures (Tamorri, 1999). The team’s ability to act collectively is critical to gaining an advantage over the opponent (Cannon-Bowers and Bowers, 2006; Lamas et al., 2014). At present, one strategy to achieve this advantage is to create a defensive imbalance by hindering the defenders’ trajectory (Vélez and López, 2010). The most effective way is to perform direct actions that cause problems to the opponent ball-handler and are accomplished through pick-and-roll at the level of collective tactics. Pick-and-roll (also known as screen-and-roll, on-ball screen, or ball screen) represents the most widespread cooperative action among the players of a high-level basketball team during offensive processes (Cárdenas et al., 1999; Vaquera et al., 2016), which is capable of placing the opponent’s defense in a more difficult situation than 1-on-1, as emphasized by most specialized literature (Ociepka, 2004; Ratgeber, 2004; Coello, 2005; Messina, 2005; Ivanovic, 2006; Harris, 2007). This tactical combination, which normally involves physical contact, is characterized by the legal interposition of an offensive player without the ball in the defensive trajectory of the offensive player in possession of the ball (Comas, 1991): a player realizes a screen (pick) for the teammate with the ball and then cuts the opponent’s area moving toward the basket (roll) to receive a pass (Koutsouridis et al., 2018). The relevance created must be analyzed and explored quickly to provide the best conditions of space on the court and obtain the best possible shooting moment (Scariolo, 2005). Therefore, a high-level basketball team must be able to execute complex actions, anticipate the development of events at the spatial and temporal levels, and make decisions quickly (Aglioti et al., 2008). All of these can be achieved either directly from the advantage created or by forcing aid, rotations, or changes that, in this way, break the defensive balance. Since pick-and-roll has become a key offensive action in recent years, teams have increased its use during both attacks and transitions and counter-attacks (Coello, 2005; Ivanovic, 2006), becoming the most important element in the strategy of many coaches (Ionescu, 2015). Therefore, studying the mechanisms underlying these strategies has become a primary goal to increase the performance of elite teams, which seems to derive not only from actions that the athlete performs but also from the observation of others (Aglioti et al., 2008). Remmert (2003), analyzing frequencies and occurrences of offensive behaviors in high-level teams, observed that pick-and-roll was the most used action to end attacks, followed by staggered screen and pick away; they are, in this order, the basic collective tactical means to obtain more points per attack. Christmann et al. (2018) studied the types of offensive actions realized in the last 2 min of 115 National Basketball Association (NBA) games and confirmed that pick-and-roll was the most used offensive action (29.1%; *n* = 290). Muñoz et al. (2015) and Nunes et al. (2016) observed that the central zone is the part of the court where pick-and-roll is most commonly implemented and the perimeter players are the most advantaged by this offensive action. These studies, therefore, support the essential role that the pick-and-roll has in basketball, fostering the great interest of the collective of coaches in this offensive action, as demonstrated by the broad range of technical publications on this subject (e.g., Coello, 2005; Filipovski, 2005; Refoyo et al., 2009; Alves, 2010; Nunes and Iglesias, 2010; Scariolo, 2015).

However, if, on the one hand, the screener interacts with the defender to free his teammate, and, on the other hand, the blocked player cannot stand still, the screener’s defender has to adjust his position continuously (Comas, 1991). Messina (2005) highlights the presence of difficulties in defending the screen because this situation is a collective work of 5-on-5, and not 1-on-1, where the offending team tries to create its advantages, while the defensive team tries to counter this. For teamwork to be effective, defenders must first have overall control of the situation where they are involved. To achieve this is very difficult, that is why players have to implement two critical behaviors during a match. On the one hand, defenders must communicate with each other (defensive communication) to obtain a positive outcome from the defense of pick-and-roll: its absence will result in shots without opposition from forward (Wootten, 2012; Wissel, 2015). On the other hand, they must carry out aggressive actions against the rival team, which are developed by technical teamwork based on the analysis of the opponent team’s capabilities. These actions, which consist of preventing the opponent from performing pick-and-roll, represent the real foundation of the defense. They are mainly implemented by the screener’s defender using his body to prevent the forward from reaching the desired space. All this requires a broad view of the game and good communication between players. Moreover, players have to analyze several aspects to make the correct decision on defending the pick-and-roll (Escuela Nacional De Entrenadores de la [FEB], 2010): (a) the place where the screen occurs (e.g., away from the basket, central or lateral); (b) characteristics of the playmaker and the screener, depending on the players involved; (c) position of the players not involved in the screen; (d) e tactical decisions of the team; (e) residual time of ball possession (e.g., less time means that the team on offense will be more aggressive). In general, defense can prevent the screen either individually (one player defends the ball handler) or collectively (through defensive aids).

Research on this area has shown several results in terms of effectiveness, defensive options, collective decision, and ways of classifying defense by both the screener’s defender and the collective decision. Authors, such as Kruger (2007) and Battaglia et al. (2009), have analyzed areas where pick-and-roll actions occur. Remmert (2003), on the other hand, describes strategies on how to defend this action of the game, while Refoyo et al. (2007) and Battaglia et al. (2009) conclude that the short defensive flash appears strongly associated with defensive success. Manzano et al. (2005) describe there are alterations in the structure of both teams in pick-and-roll actions.

As we can see, although research emphasizes the impact of several factors on the effectiveness of offensive and defensive tactics, there is a scarcity of studies on the frequency of pick-and-roll use, court zones where it occurs, and tactical sequences of teams. Furthermore, no study has investigated the offensive and defensive dynamics on the basis of pick-and-roll, which are implemented by an elite team during an entire season of the *Asociación de Clubes de Baloncesto* (ACB) league. Finally, to our knowledge, no study has evaluated the pick-and-roll from the perspective of elite team coaches.

The field of physical activity and sport is rich in statistics from different sources (e.g., sports press, professional leagues, and sports organizations), which provide information both at a macro level (e.g., defining team scores and trajectories) and at a more micro-level (e.g., determining the characteristics of each player, rebounds, and assists). However, they do not allow for capturing the reality of a basketball game. In this sense, the paradigm of mixed methods permits the analysis of high-performance offensive and defensive tactical behaviors from a systemic-ecological perspective (Anguera et al., 2018), fostering an explanatory scientific knowledge of behaviors emerging during the pick-and-roll within the competitive environment of the playing court. In particular, the observational methodology, considered as a mixed method itself (Anguera et al., 2017, 2018), is suitable for the analysis of collective sports as demonstrated by numerous studies in this field (e.g., basketball, Garzón et al., 2011; handball, Lozano et al., 2016; rugby, Passos et al., 2012; volleyball, Molina et al., 2008). This methodology, indeed, detects levels or dimensions in which an individual’s participation in collective sport games occurs (Martín Acero and Lago, 2005), allowing for the construction of an objective model for gathering information on key elements of the pick-and-roll that can be quantifiable in a consistent and significant way (Nevill et al., 2008). Therefore, observing the offensive and defensive dynamics of the pick-and-roll in high-level teams of the ACB league can be useful to increase the knowledge of underlying cognitive processes that emerge in a field that is still poorly studied. In particular, the assessment of the perspective of high-level coaches on this issue can provide important information on teamwork and players to improve the effectiveness of the decision-making process and structure actions aimed at achieving a positive outcome during a match. The main purpose of this study is to analyze deeply interviews of high-level coaches to investigate their approaches on offensive and defensive behaviors that develop within the pick-and-roll, paying particular attention to their viewpoints on the dynamics that emerge in an elite basketball team during an entire season of the Spanish ACB league.

## Materials and Methods

### Design

We applied a mixed-method approach, integrating qualitative and quantitative elements, through the *connecting* option proposed by Creswell and Plano Clark (2017), which consists of building one dataset on top of the other, obtaining full integration (Anguera et al., 2017). To achieve this, the indirect observational methodology was used by realizing a non-participant systematic observation of verbal behaviors of high-level coaches regarding the offensive and defensive aspects of pick-and-roll in high-level teams emerging during the ACB league (Anguera and Hernández-Mendo, 2016; Anguera et al., 2017). According to observational designs described by Anguera et al. (2018), the study was performed using a nomothetic/punctual/multidimensional (N/P/M) design. It was *nomothetic* because the observation unit consisted of an in-depth individual interview with six high-level basketball experts. It was *punctual* (with intra-sessional following) regarding temporality of the evaluation, as each participant was observed through a single interview session analyzing the succession of behaviors. Finally, it was *multidimensional* in terms of the dimensionality of behaviors analyzed, as we observed different verbal dimensions related to the offensive and defensive components of pick-and-roll from the perspective of high-level coaches. This multidimensionality of the participants’ responses is reflected in the *ad hoc* indirect observation tool that was built for this study.

### Participants and Materials

First, the research was evaluated and approved by the Ethics Committee for Clinical Investigations of the Sports Administration of Catalonia (no. 24/20118/CEICEGC). It was presented to the participants and carried out after they accepted and signed the informed consent concerning audio recording, interview collection, and use of the results. To this end, we followed the guidelines of the project approved by the ethics committee and the ethical principles related to the Psychologist’s Code (General Council of Official Colleges of Psychologists, 2010) and the Declaration of Helsinki.

An interview was conducted on six high-level coaches recruited as follows: two of them were head coaches of the Unicaja Málaga team in the 2010–2011 season (coaches 1 and 3); three were assistant coaches of that team in the same period (coaches 2, 4, and 5); one was a Spain basketball men’s national selector (coach 6) who was chosen because of his neutral position concerning the results obtained by the team from the period considered.

We have chosen to observe the ACB (*Asociación de Clubes de Baloncesto*) league for its competitiveness and the quality of players and coaches. Unicaja belongs to a small group of ACB teams that have already won the league (2005–2006), the King’s Cup (2004–2005), and the Eurocup (2016–2017). In the season analyzed, this team played in the EuroLeague. Of 20 players on the team, 13 were internationals representing their countries and 4 have played in the NBA.

We analyze this season for two other circumstances: first is because in the middle of the season (50% of league games) the team changed coach, which could mean tactical changes and modifications on how to use and defend the ball screen; second is because, in that season, there was a change in regulation, fundamentally in the lines of the court, which required a different analysis of previous studies with a different disposition of the lines of the game.

Our observational record focused on all matches of the same team, Club Unicaja Baloncesto, throughout the season, with data from two matches with each of their rivals corresponding to two rounds in the regular league, and with a different coach in each of these rounds.

All those interviewed have been Unicaja technicians, have been part of the technical staff of the Spanish team, have achieved a national or international title as coaches, and are currently active.

### Instruments

#### Observational Instrument

We structured an interview to collect opinions of the high-level coaches (*n* = 6) on the offensive and defensive dynamics of pick-and-roll (*n* = 2224) that emerged during the analysis (Nunes, 2020) of 34 matches played by the team Club Baloncesto Málaga (Unicaja Málaga) in the 2010–2011 season of the ACB league. The interview consisted of 16 questions (Table 1) divided into eight general pick-and-roll questions and eight questions about the Unicaja team.

**TABLE 1:** In-depth interview questions for basketball experts about the pick-and-roll in the analysis (Nunes, 2020) of Club Baloncesto Málaga (Unicaja Málaga) of the *Asociación de Clubes de Baloncesto* (ACB) league.

Introductory question (reality at the time to contextualize; interviewee comfort; cognitive and emotional filter).
1. There is an average of 33 pick-and-rolls (PR) and 4 simulations performed per team and per match. Did you expect this result? Why?
2. 72% (*n* = 1604) of the pick-and-rolls performed are executed in the initial phase of the offensive system of the team. Is it a number that surprises you? Why?
3. The figure shows the distribution of the pick-and-rolls made according to the area of the court. Did you expect this outcome? Why?
4. After the pick-and-roll, we observed to which side the player dribbles the ball. 54% of the time, he dribbles to the right and 46% to the left. Do you consider this relationship to be the expected one, or did you think there would be a greater difference? Why?
5. After the pick-and-roll, 51% of the actions registered ends in a shot. What do you think of this percentage of shooting stock? Is it the best resource to achieve an optimal shooting position or to create the options to achieve it? Why?
6. However, only 2% of the above actions are immediate shots, and only 30% (*n* = 16) of this value is converted into points. What do you think of these figures? What strategies do you think could improve these percentages?
7. We found a variety of offensive combinations after the pick-and-roll. The most frequent action (12% of the total; *n* = 256) was: ball handler dribbles, passes to another teammate and the latter makes another pass (B1BPTRP). What do you think?
8. If we analyze the defensive phase of the pick-and-roll, we see how the defender of the ball handler escapes the screen on 28% of the occasions. What do you think of this percentage? What can defenders do to improve their response to this technical-tactical action?
9. In Unicaja (10/11), 73% of the team’s offensive actions after a time-out are performed through a pick-and-roll. Do you consider that, after a time-out, the initial offensive resource should always go through a pick-and-roll? Why?
10. In Unicaja (10/11), 51% of the actions after the pick-and-roll have involved a third player in addition to the ball handler (B1) and the screener (B2). What do you think of these results?
11. In Unicaja (10/11), *pick* and *repick* (re-screen) were observed in 53 records (5% of the total pick-and-rolls). What do you think of this number of *repicks*? Why?
12. In Unicaja (10/11), 15% of the pick-and-roll was used with the intervention of a second screener (used or not). *Horns*’ tactical work has been a classic in modern basketball. Would you expect another result? Why?
13. In Unicaja (10/11), we observed that the team performed more pick-and-roll actions in the possessions where they were winning the match (524 possessions) compared to the possessions where the team was losing (506 possessions). Is the strategy of using pick-and-roll conditioned by the result on the scoreboard? Is it a deliberate resource, or is it a random piece of information?
14. In Unicaja (10/11), a 2 on 1 trap has been made in 8% (*n* = 95) of the observed defenses, and a defensive change has been made in 19% (*n* = 216). Do you think they are good defensive options? Why?
15. In Unicaja (10/11), the main defensive responses of the ball handler’s defender have been to *chase* (38%; *n* = 439), *over the top* (23%; *n* = 261), and *under the screen* (18%; *n* = 205). For you, are the best options to defend the pick-and-roll? Why?
16. In Unicaja (10/11), the main defensive responses of the screener’ defender have been *open* (41%; *n* = 466), *show* (36%; *n* = 411), and *hedge* (15%; *n* = 176). For you, are the best options to defend the direct pick-and-roll? Why?
Final question (reality at that moment to contextualize; interviewee comfort; cognitive and emotional filter).

Individual interviews were recorded in a room that guaranteed privacy and reduced environmental noise by placing a device at a suitable distance from the interlocutor. We performed the verbatim transcription of recorded audio materials for a total of six interviews that were systematically observed (indirect observation of the answers of each one).

The observation of high-level coaches’ evaluations of the pick-and-roll was carried out by narrative content analysis of the responses, reflecting their positions and emotions on the topic (Campo et al., 2019). To this end, we used the *ad hoc* indirect observation tool, which combines field formats with category systems, two tools from observational methodology (Anguera et al., 2018). It is composed of two main dimensions or criteria, generic answer and answer justification, divided into 4 and 15 subdimensions, respectively, and the results are in line with the objectives of the study (Table 2). This new instrument detects 41 verbal behaviors to analyze the offensive and defensive aspects of pick-and-roll. By observation of these behaviors, it is possible to know the effectiveness of the dynamics characterizing the pick-and-roll in high-level basketball according to the opinions of the high-level coaches. The criteria characterizing the instrument are applied to the coaches’ answers; a sentence is assumed as the analysis unit based on a syntactic criterion, not considering verbalizations that do not provide any concept related to the questions. Each criterion gives rise to respective category systems fulfilling the condition of exhaustiveness and mutual exclusivity (E/ME; Anguera et al., 2018).

**TABLE 2:** Indirect observation tool to analyze the narrative content of responses on the offensive and defensive aspects of pick-and-roll.

Dimension	Sub-dimension	Category	Code	Recoding
General answer (D1)	Significant content (D11)	Positive or favorable	D111	General and favorable evaluation (R1)
		Positive or favorable evaluation reinforced	D112	
		Negative or unfavorable evaluation	D113	General and unfavorable evaluation (R2)
		Negative or unfavorable evaluation reinforced	D114	
		Neutral evaluation	D115	General and neutral evaluation (R3)
		Neutral evaluation reinforced	D116	
	Non-significant content (D12)	Non-significant evaluation	D121	
	Emotional content (DI3)	Emotional or expectation evaluation	D131	
		Emotional or expectation evaluation reinforced	D132	
	Limiting content (DI4)	Conditional or limiting valuation	D141	
		Conditional or limiting valuation reinforced	D142	
Answer justification (D2)	Team planning argument (D21)	Roster	D211	
	Game time argument (D22)	Regular time	D221	
		Action time	D222	
	Game area argument (D23)	Regular area	D231	
		Action area	D232	
	Regulatory argument (D24)	Regulations	D241	
	Result-oriented argument (D25)	Scoreboard	D251	
	Technical argumentation (D26)	Offensive technique	D261	
		Defensive technique	D262	
	Individual tactics argumentation (D27)	Individual offensive tactics	D271	
		Individual defensive tactics	D272	
	Team tactics (D28)	Collective offensive tactics	D281	
		Collective defensive tactics	D282	
	Reasons leading to decision making (D29)	Decision making	D291	
	Physical argumentation (D210)	Individual physic	D2101	
		Collective physic	D2102	
	Psychological argumentation (D211)	Psychological	D2111	
	Team argumentation (D212)	Main team observed (Unicaja)	D2121	
		The other team observed	D2122	
		The other team no observed	D2123	
	Player/coach argumentation (D213)	Player/coach of the main team observed (Unicaja)	D2131	
		Player/coach of the other team observed	D2132	
		Player/coach of the other team no observed	D2133	
	Comparisons (D214)	Inter-team	D2141	
		Inter-player	D2142	
		Inter-coach	D2143	
		Inter-competition	D2144	
		Inter-season	D2145	
	Different support (D215)	Visual support	D2151	
		Numerical or statistical support	D2152	

#### Recording Instruments

The recording instrument used was the voice note application of iPhone 4 and 6S mobile phones (Apple^®^) to obtain the audio recording. We used Word files to transcribe the interviews verbatim, which were subsequently reported in Excel file and divided into speaking turns referred to the interviewer and each coach, respectively. Finally, we assessed data quality by calculating Cohen’s κ (Cohen, 1960) coefficient with the GSEQ5 v. 1.23 computer program (Bakeman and Quera, 2011).

### Procedure

Coaches’ verbal behaviors related to offensive and defensive tactics for pick-and-roll were analyzed and coded by applying the indirect observation tool to every answer in the interviews. Before full data set coding, an expert observer recorded 15% of the total material following Krippendorff’s (2018) recommendation. Intra-observer reliability, the agreement level of an observer in coding of the same material at two different times (in this study after a week), was calculated with GSEQ5 (Bakeman and Quera, 2011). The resulting κ was 0.97, corresponding to almost perfect reliability (for κ ≥ 0.81; Cohen, 1960), which guarantees the interpretative rigor of the coding process. After passing data quality control, the indirect observation instrument was applied to the rest of the interviews. We obtained a code matrix for each question that included coded responses of the six coaches. Each line of each matrix expressed co-occurrent and event-based codes (Bakeman, 1978; see the example in Table 3).

**TABLE 3:** Example of code matrix related to question 4.

Coach	Sentence	Code						Recoding
C1	S1	D141	D261	D271	D2142			R1
	S2	D142	D115	D2152	D261			
C2	S1	D111						R2
	S2	D131	D2152					
	S3			D2142	D232	D2152	D291	
C3	S1	D111	D2152	D261				R1
	S2	D112	D261	D271				
	S3	D141		D261	D271	D2152	D281	
	S4			D261	D271			
	S5	D112	D271					
C4	S1	D131	D2152	D291	D282			R2
	S2	D121						
	S3			D282				
	S4			D282	D262	D2102		
	S5			D282				
	S6			D2132	D261			
	S7			D2142				
C5	S1	D115						R1
	S2			D261	D232	D281		
	S3	D116	D2152					
	S4	D141	D261	D2152	D2142			
C6	S1	D111	D261	D281				R2
	S2	D131	D261	D291	D271			

*C, coach; S, sentence.*

To investigate the relationship between positive/negative perceptions of the six coaches and offensive/defensive tactical aspects of pick-and-roll in the 2010–2011 ACB league presented in the questions, we performed a recording process by grouping the data of some basic categories into macro-categories with more global characteristics according to the molar level of granularity in the observation defined by Schegloff (2000). Based on an evaluation criterion, we structured three new macro-categories: R1, R2, and R3. R1 expresses the general and favorable evaluation by the coaches of pick-and-roll aspects presented through questions. It results from the aggregation of categories D111 (positive or favorable evaluation) and D112 (positive or favorable evaluation reinforced). R2 corresponds to a general and unfavorable evaluation of the data presented through a question based on the aggregation of categories D113 (negative or unfavorable evaluation) and D114 (negative or unfavorable evaluation reinforced). Finally, R3 expresses a general and neutral evaluation of coaches of the pick-and-roll dynamics presented through a question. It results from the aggregation of the categories D115 (neutral valuation) and D116 (neutral evaluation reinforced). Given the neutral nature of these data, we decided to focus only on R1 and R2.

### Data Analysis

To achieve the objectives of the study, we performed a polar coordinate analysis, which was developed by Sackett (1980) and subsequently improved by Anguera (1997). The main objective is to obtain a complete map of interrelationships among textual units (indirect observation) and represent it graphically through vectors. This quantitative analytical technique identifies statistically significant relationships among different behavior codes, specifically, between the behavior of interest (*focal behavior*), which is considered central or core, and other associated behaviors (*conditional behaviors*). This technique offers wide possibilities of use, as proven by numerous studies on sports sciences (e.g., Castañer et al., 2017; Tarragó et al., 2017; Maneiro and Amatria, 2018; Prudente et al., 2018; Vázquez-Diz et al., 2019; Jiménez-Salas et al., 2020; Pastrana-Brincones et al., 2021).

The polar coordinate analysis involves initial calculation of adjusted residuals by sequential lag analysis (Bakeman, 1978; Bakeman and Quera, 2011), which a researcher performs to learn how behavior works at an early level of abstraction. The succession of sessions over time drives researchers to delve deeper in the search for common elements that occur with a greater or lesser degree of presence and consolidation and that connect sequentially with each other. This analysis allows for studying processes over time, giving a dynamic view of how the behavior patterns obtained allow researchers to know about the evolution produced, with elements of these patterns likely to be modified, and with connections among them that may remain stable or evolve (Anguera et al., 2021).

The adjusted residuals obtained by the lag sequential analysis are the starting data of the polar coordinate analysis. These residuals, calculated prospectively (from lag + 1 to lag + 5) and retrospectively (from lag −1 to lag −5), are standardized and used to calculate the *Z*_*sum*_ statistics proposed by Cochran (1954) as relative indices of sequential dependence that allows to build a vector map showing the statistical relationship between the focal and conditional behaviors. The Prospective *Z*_*sum*_ (*Z*_*sum*_*P*) and Retrospective *Z*_*sum*_ (*Z*_*sum*_*R*) values are reported on the *X* and *Y* axes, respectively, defining the four quadrants of the vector map where the focal behavior represents the zero point. Radius calculation (radius = $\sqrt{{\left(Z_{s\,u\,m}\,P\right)}^{2}+{\left(Z_{s\,u\,m}\,R\right)}^{2}}$) makes it possible to determine the strength of the relationship, which is significant for values greater than 1.96 and *p* < 0.05, while the angle $a\,r\,c\,s\,i\,n\,e\,\varphi =\frac{Z_{s\,u\,m}\,R}{r\,a\,d\,i\,u\,s}$ expresses the nature of the relationship itself. The value of the latter (φ) is transformed according to the quadrant in which the vector is located.

Quadrant I (+ +) shows the reciprocal activation between the focal and conditional behaviors. In quadrant II (− +), the focal behavior inhibits the conditional behavior; simultaneously, the latter activates the former. Quadrant III (−−) highlights the mutually inhibitory relationship between focal and conditional behaviors. Finally, the vector positioned in quadrant IV (+) shows that the focal behavior activates the conditioned one; at the same time, the latter inhibits the former. In this study, the macro-categories R1 and R2 were assumed as focal behaviors, while categories of the dimension Justification of Response were selected as conditional behaviors to analyze the dynamics between the perceptions of coaches and the tactical aspects of pick-and-roll emerging in each interview question. The HOISAN program (v. 1.6.3.4; Hernández-Mendo et al., 2012) made it possible to perform these calculations by obtaining parameter values and vectors. After, these vectors were optimized graphically using the R program (Rodríguez-Medina et al., 2019). In this study, we considered the coaches’ perspectives (the general and favorable evaluation [R1] and the general and unfavorable evaluation [R2]) on the pick-and-roll aspects presented through each question as focal behaviors. On the other hand, the verbal behaviors related to answer justification were assumed as conditional behaviors. The polar coordinate analysis and vectorial maps were performed and used with HOISAN and R by considering five lags for *Z*_*sum*_ R (from lag −5 to lag −1) and *Z*_*sum*_ P (from lag + 1 to lag + 5).

## Results

**FIGURE 1:**
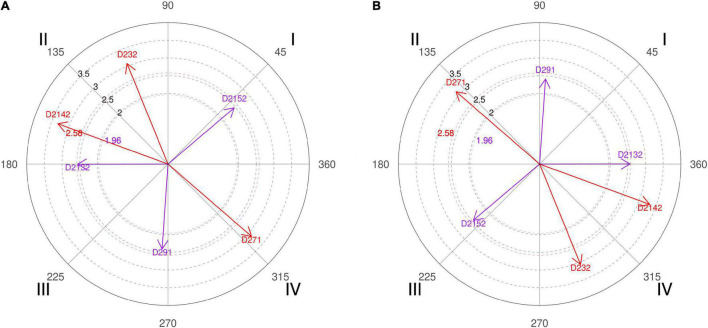
**(A,B)** Relationship of conditioned behaviors that present a significant relationship with the focal behaviors “favorable evaluation” and “unfavorable evaluation” analyzing question 4 of the in-depth interviews. By way of example, in “favorable evaluation,” a total of 6 significant vectors were detected, of which 1 was in quadrant I, 2 were in quadrant II, 2 were in quadrant III, and 1 was in quadrant IV. In “unfavorable evaluation,” a total of 6 significant vectors were detected, of which 2 were in quadrant I, 1 was in quadrant II, 1 was in quadrant III, and 2 were in quadrant IV.

**FIGURE 2:**
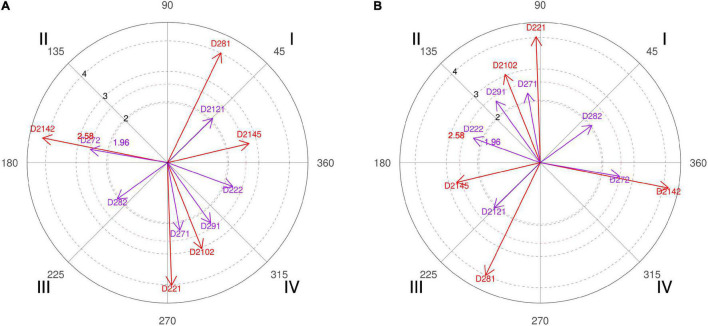
**(A,B)** Relationship of conditioned behaviors that present a significant relationship with the focal behaviors “favorable evaluation” and “unfavorable evaluation” analyzing question 5 of the in-depth interviews. In “favorable evaluation,” a total of 11 significant vectors were detected and in “unfavorable evaluation,” a total of 11 significant vectors were detected.

**FIGURE 3:**
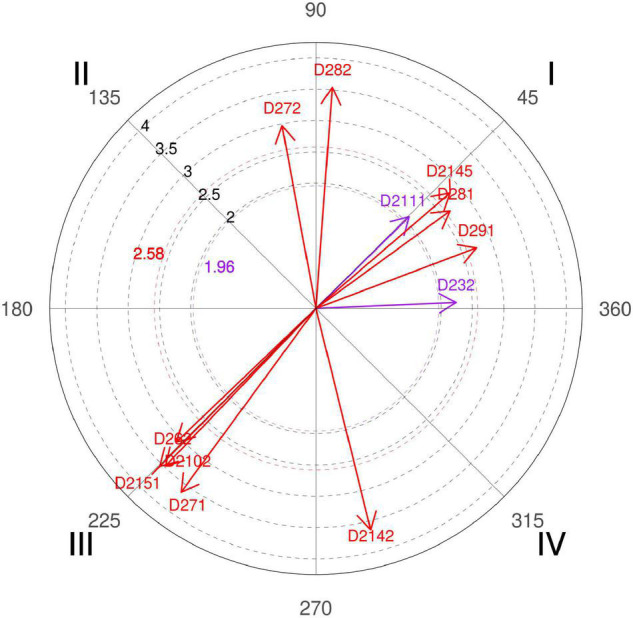
Relationship of conditioned behaviors that present a significant relationship with the focal behavior “unfavorable evaluation” analyzing question 8 of the in-depth interviews. In “unfavorable evaluation,” a total of 12 significant vectors were detected.

**FIGURE 4:**
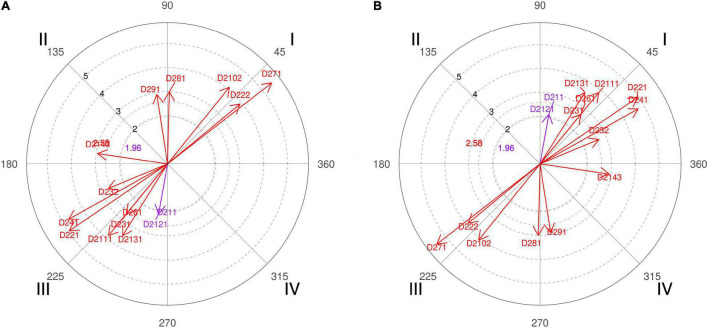
**(A,B)** Relationship of conditioned behaviors that present a significant relationship with the focal behaviors “favorable evaluation” and “unfavorable evaluation” analyzing question 9 of the in-depth interviews. In “favorable evaluation,” a total of 15 significant vectors were detected, and in “unfavorable evaluation,” a total of 15 significant vectors were detected.

**FIGURE 5:**
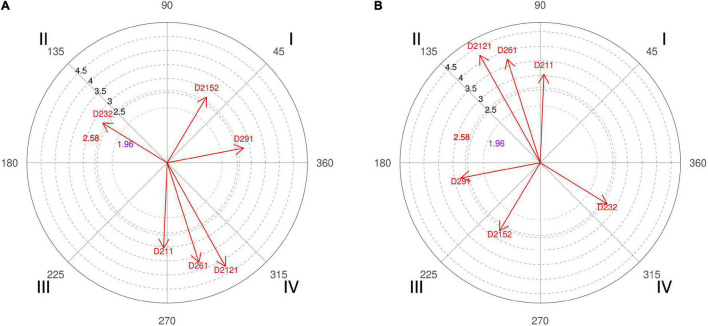
**(A,B)** Relationship of conditioned behaviors that present a significant relationship with the focal behaviors “favorable evaluation” and “unfavorable evaluation” analyzing question 10 of the in-depth interviews. In “favorable evaluation,” a total of six significant vectors were detected, and in “unfavorable evaluation,” a total of six significant vectors were detected.

**FIGURE 6:**
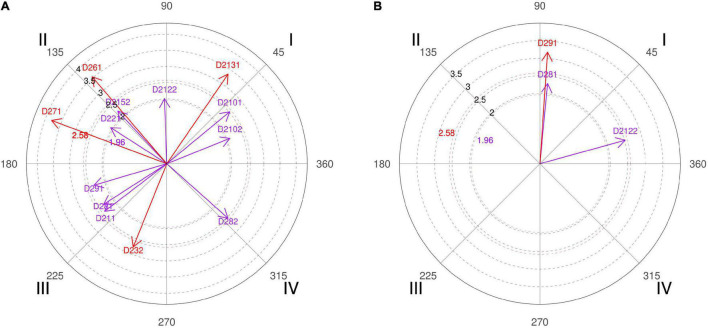
**(A,B)** Relationship of conditioned behaviors that present a significant relationship with the focal behaviors “favorable evaluation” and “unfavorable evaluation” analyzing question 11 of the in-depth interviews. In “favorable evaluation,” a total of 13 significant vectors were detected, and in “unfavorable evaluation,” a total of three significant vectors were detected.

**FIGURE 7:**
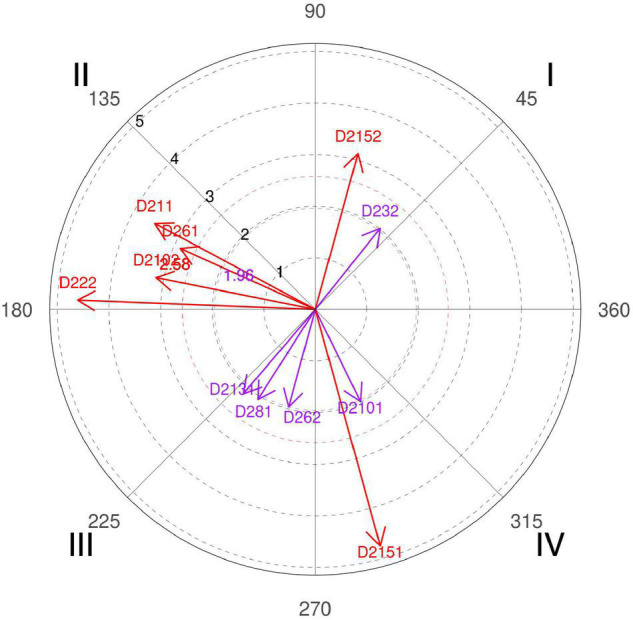
Relationship of conditioned behaviors that present a significant relationship with the focal behavior “favorable evaluation” analyzing question 12 of the in-depth interviews. In “favorable evaluation,” a total of 11 significant vectors were detected.

**FIGURE 8:**
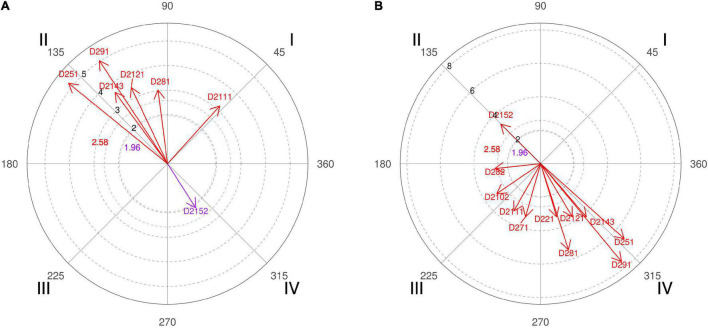
**(A,B)** Relationship of conditioned behaviors that present a significant relationship with the focal behaviors “favorable evaluation” and “unfavorable evaluation” analyzing question 13 of the in-depth interviews. In “favorable evaluation,” a total of 7 significant vectors were detected, and in the “unfavorable evaluation,” a total of 11 significant vectors were detected.

**FIGURE 9:**
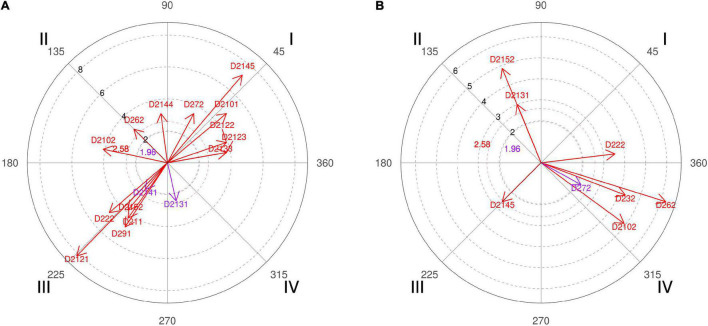
**(A,B)** Relationship of conditioned behaviors that present a significant relationship with the focal behaviors “favorable evaluation” and “unfavorable evaluation” analyzing question 14 of the in-depth interviews. In “favorable evaluation,” a total of 16 significant vectors were detected, and in “unfavorable evaluation,” a total of eight significant vectors were detected.

**FIGURE 10:**
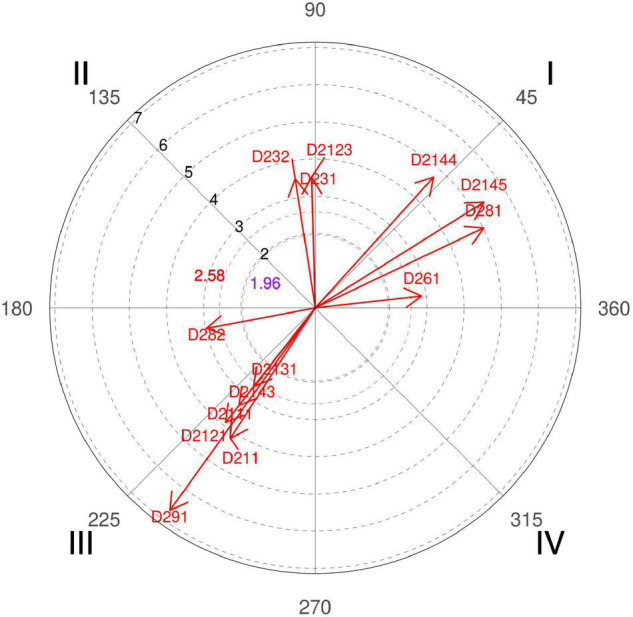
Relationship of conditioned behaviors that present a significant relationship with the focal behavior “favorable evaluation” analyzing question 15 of the in-depth interviews. In “favorable evaluation,” a total of 14 significant vectors were detected.

**FIGURE 11:**
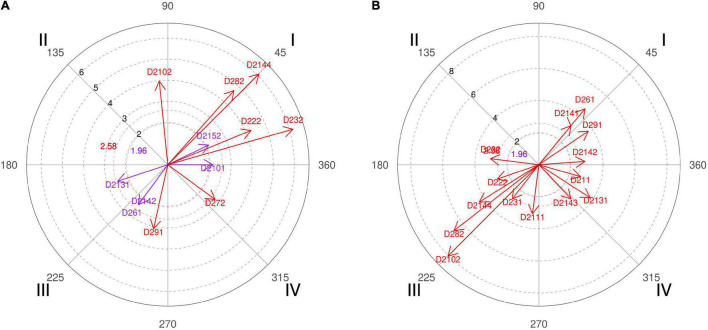
**(A,B)** Relationship of conditioned behaviors that present a significant relationship with the focal behaviors “favorable evaluation” and “unfavorable evaluation” analyzing question 16 of the in-depth interviews. In “favorable evaluation,” a total of 12 significant vectors were detected, and in “unfavorable evaluation,” a total of 14 significant vectors were detected.

To achieve the objective of this study (to analyze the pick-and-roll in an elite team during an entire ACB league season from the perspective of high-level coaches), we focused on favorable and unfavorable perceptions that the six coaches showed about the offensive and defensive dynamics in the 2010–2011 season of Unicaja Málaga. Figures 1–11 show the results derived from the polar coordinate analysis where each vectorial map represents the statistically significant association between positive and negative evaluations by the coaches of pick-and-roll aspects (*focal behavior*) and verbal behaviors justifying their perspective (*conditional behaviors*) (codings related in the Figures can be identified in Table 2). We will briefly discuss only vectors with a length greater than 1.96 (*p* < 0.05) that express activations between focal and conditional behaviors in each vectorial map.

## Discussion

Our study aimed to objectively analyze the offensive and defensive behaviors that emerge in the pick-and-roll through the viewpoint of a group of high-level coaches, with particular attention to the dynamics required in a basketball elite team during an entire season of the Spanish ACB league.

This article presents, for the first time in the literature, an analysis, with a mixed-methods approach, of expert reflections of a group of elite basketball coaches on the importance of the pick-and-roll in the development of the game, with special attention to the offensive and defensive tactical processes and taking into account both participation of players in this tactical action, as well as the situational aspects that have an influence (such as field area, playing time, and result).

From the qualitative data collected from the interviews, a quantitative polar coordinate analysis was conducted after coding. It allowed us to know the associative relationships between each focal code and corresponding codes of conditioned behaviors. This extremely rigorous and robust analysis provided nuances of the intensity of the relationships (vector length) and their nature (angle of the vectors and their corresponding positions in the quadrants), which is new in elite basketball.

We will develop this discussion following the coaches’ responses to the questions raised (Tables 1, 2) specifically related to the importance of pick-and-roll in tactical approaches of the game (questions 4, 5, 8, 9, 10, 11, 12, 13, 14, 15, and 16). In these cases, in which all the answers were equal or had no significant results, the analysis was not performed: questions 1 and 6 (all answers were R2), questions 3 and 7 (all answers were R1), and question 2 (results were not significant).

*Question 4: After the pick-and-roll, we observed to which side the  player  dribbles  the  ball, and 54% of the time, he dribbles to the right and 46% to the left. Do you consider this relationship to be  the  expected  one,  or  did you think there would be a greater difference? Why?*

According to Perše et al. (2009), there are two very important elements in this offense, which are: the motion of the ball handler (right–left), and motions of the players without the ball. Obviously, this offensive action is characterized by continuous motion.

Below are some of the comments expressed in the interviews with experts in response to question 4, reinforcing their explanation with the results of the polar coordinate. The responses varied, since the group of coaches, coaches 2, 4, and 6, expected a higher percentage of screens to the right side, and the group of experts, coaches 1, 3, and 5, was not so surprised by the result obtained. Coach 2 defended that in basketball there, is a “greater number of ball handlers with the right and that everything that facilitates the progression of the ball to the right side implies generating advantages.” Even coach 4 went further with his explanation, arguing that at the time of making the scouting of rival teams he was thinking about the characteristics of the opponents (polar coordinate Figure 1B, quadrant I, category D2132) who practice a *double pick-and-roll/horns* and “the defense sends directly to the left side (the player’s weak hand) or where we have defensively the power forward player because normally these players manage to defend better than the center players.”

However, the study by Stavropoulos and Stavropoulos (2020) indicates that, when the ball handler chooses the left direction of motion after a slip pick, pick and pop, and stretch, the trap on the top of the key and left side of the court achieves better effectiveness than the right side. It was also observed that the ball handler prefers to move to the right side toward the basket rather than to the left on the top of key. Presumably, this happens because majority of ball handlers use their right hand as their dominant one. Furthermore, an important finding is that the ball handler prefers to move to the left when he is on the right side. This probably happens because his space is restricted if he moves to the right toward the baseline; additionally, in this restricted space, there are already defensive players. For these reasons, if he moves to the left, he is given more offensive options. The opposite is true for the left side, where the ball handler prefers to move to the right for the same reasons that apply for the right side. This is in line with Van Maarseveen et al. (2018) who claimed that players use their non-dominant hand on the right side of the court and the dominant hand when playing on the left.

Battaglia et al. (2009), by conducting an analysis on pick-and-roll actions that occurred in the defensive court during ACB league matches (season 2007/2008), observed that on 770 screens, 51.6% was achieved in the central zone with a defensive success of rate 44.9%, and that 48.4% was achieved by bounds with a defensive success rate of 58.7%. The authors concluded that defense acts better on bound-oriented screens, as confirmed by the study of Kruger (2007).

Gómez-Ruano et al. (2015) and Vaquera et al. (2016) confirmed in their studies that there is less offensive effectiveness when the screener establishes a screen from the central zone toward the lateral zones and greater effectiveness when the screen is established from the lateral zones toward the central zone or toward the baseline. Uxía et al. (2012) and Ionescu (2015) argued that teams study screening tactical options to favor the dominant hand of players, which is normally the right.

These also result with adjusted percentages and coach 6 affirms that “most of the players are right-handed” but he also explains two factors that can influence the offensive decision: “One, aside from left-handers who are not a decisive factor, there are right-handed players who go to the left better than to the right, especially to stop and shoot rather than to penetrate; and two, when the defense passes behind almost everyone re-screen (*repick*) that makes the player end up going to the left.”

Elite teams train and decide how help defense will be on the weak side or the strong side, and establish a table of possible rotations, always thinking about the capabilities and characteristics of both teams.

Coach 3 states that there are right-handed players who are very good shooters and who prefer to move to the left since “they perform the static shot better.” Basketball is in constant development, adapting to elite performance, and “with time the players are better and they dominate both hands more” (coach 5).

*Question 5: After  the pick-and-roll, 51% of actions registered ends  in a shot. What do you think of this percentage of shooting statistic?  Is  it  the  best resource to achieve an optimal shooting position or to create options to achieve it? Why?*

Our study (Nunes, 2020) states that 51% of actions registered after a screen ended in shooting. Also, for Romero (2008), it is an action that obtains effective results 50% of the time. In the end, what all basketball coaches look for is success of the offensive phase, and this is determined by the effectiveness of shots (Ibáñez et al., 2009; Serna, 2014; Serna et al., 2017). Consequently, players have to be trained in executing a ball screen by learning to read the defense properly (Remmert and Chau, 2019).

The Spanish national head coach, coach 6, explains that “the magic of screening is precisely attracting two players at a given moment and having the speed of passing the ball to the teammate who has become free” and that is why a high percentage of shots are achieved after this technical-tactical action. It is a phrase that explains well what basketball professionals look for from the pick-and-roll, and the polar coordinate also expresses that for the interviewed coaches, the richness of the offensive tactic of screening allows for creation of several reliable options to hinder the opponents’ defensive work and achieve a sum of points on the scoreboard (Figure 2B, quadrant III, category D281).

Coach 1 clarifies that if most screens are made in the beginning of possession, it serves to find the best shot option among the player with the ball, the screener, and a third player who can intervene in the offensive action, but the pick-and-roll made in the end of possession is made as a last resort to seek an immediate shot option. The idea of the head coach is reinforced by the result of the polar coordinate performed with coding data of the interviews carried out with basketball experts, where they support the idea that decision-making in pick-and-roll is conditioned by the moment of its performance (Figure 2A, quadrant IV, categories D222 and D291).

*Question 8: If  we  analyze  the  defensive  phase  of  the  pick- and-roll , we  see  how  the  defender  of  the  ball handler escapes the  screen  on  28%  of  the  occasion. What  do you think of this percentage?  What  can defenders do to improve their response to this technical-tactical action?*

Pick-and-roll and its variations (pop, slip, and stretch the trap) create such confusion to the defense that hardly any other offense creates, the result of which is that spying by opponent coaches becomes difficult (Stavropoulos and Stavropoulos, 2020).

Most of the elite coaches interviewed think that it is a bad defensive result, and this fact clearly explains “why screening is still such a used and effective action” (coach 6).

However, this result has had several readings, since, offense-wise, coach 3 affirms that “if the ball handler does not lead his defender to the screen, there is no effective screen, or if the big one does not place the right screen angle and at the right height the defender will also be able to escape the screen.” However, there is also the merit of the defender, since “when a screening occurs seven meters from the basket, a player normally in 1 × 1 defends a little far, half arm or even sometimes one arm, but to pass the screen and to escape you have to stick to the man with the ball, and it is a cause-effect reaction that sometimes does not occur because the center warns the screen late, or because the little one does not immediately follow the defensive call of the center to the jump reaction according to the defensive tactic planned for the man with the ball” (coach 6). A fact confirmed by the polar coordinate (Figure 3, quadrant I, categories D281, D282, and D232).

In addition to distance control, also defended by coach 2, of technical aspects that involve the action, tactical options of each team, and scouting carried out previously, coach 4 also adds that to optimize this percentage “The first thing is to improve the communication signals between teammates to be prepared because if they are ready in advance it is easier.” Authors Stavropoulos and Stavropoulos (2020) clearly explain that the pick-and-roll is an offense without verbal signals, and that the defense has that weapon in its hand, communication, since it can be adapted momentarily to the opponent’s screen action.

The main thing is the will that is put into carrying out a defensive action and the study by Christmann et al. (2018) support the idea that in 65.7% of screens analyzed, it is verified that defensive interveners of a screen have expressed a type of moderate or no defensive pressure in said situation. A fact corroborated by Remmert and Chau (2019) affirms that ball screens are most successful when defenders act passively (remaining in the screens).

Also, the polar coordinate of the interviews expresses the idea that if defenders bet on an intense defensive attitude, are aggressive in their gestures, and have the psychological harshness of wanting to defend, then these are very valid and simple arguments to make pick-and-roll a more efficient action (Figure 3, quadrant I, category D2111).

*Question 9: In  Unicaja  (10/11), 73%  of  the team’s offensive actions  after  a  timeout  are performed through a pick-and-roll. Do  you  consider  that,  after  a  time-out,  the  initial  offensive resource should always go through a pick-and-roll? Why?*

The research available on this subject is very scarce; however, the study of Sampaio et al. (2013) shows that results have shown that teams who called a timeout were able to increase points scored immediately after. In fact, after this 60-s break, players may perform better because of physiological recovery; however, their opponents also had the same recovery opportunity and performed worst (receive points). Nevertheless, physiological recovery may be determinant when interacting with technical (Lyons et al., 2006) and tactical performances (Davey et al., 2002; Royal et al., 2006).

The current results suggest that coaches should examine offensive and defensive performances when considering whether to call a time-out. Also, the results point out the importance of offensive performances and their effects according to game period and momentary differences in score (Gómez-Ruano et al., 2011).

Manzano et al. (2005) stated that screens are often used in situations where the disadvantage in the scoreboard of the one who uses them is considerable considering the time remaining and tends to risk, more than usual, facilitating and building simple attack situations with this option.

Therefore, in addition to confronting the experts with the data obtained, we also asked if they consider that after a timeout, the initial offensive resource should go through a screen. Four of the six coaches interviewed stated that this should not always be the case and even shown surprise at the high result achieved. In their justifications for such a percentage, we emphasized that “the players of that season liked to play the screens a lot and in the end, we ended up playing that resource more” (coach 5). A fact also confirmed by the polar coordinate of the interviews (Figure 4B, quadrant I, categories D211, D2121, and D2131) where strategies implemented to the players of the squad and its features are adapted. Unicaja’s coaches commented that “you can play other kinds of situations that also make your team not so predictable because the coaches of the team who is defending say during downtime, be careful that they are going to play a screen, and the players are already ready for it.” That’s why collective offensive tactics have enormous weight on professional teams (Figure 4B, quadrant III, category D281).

The polar coordinate showed that time is a key aspect for coaches (Figure 4B, quadrant III, category D222; quadrant I, category D221), since “when you have the entire possession time because it always gives you time to make a pick-and-roll. Timeout gives you time to think, you can attack the worst defender of the small players, the worst defender of the big players, or think which side to attack” (coach 4).

*Question 10: In  Unicaja  (10/11),  51%  of  actions  after  the pick-and-roll  have involved a third player in addition to the ball handler  (B1)  and the screener (B2). What do you think of these results?*

In the study of Marmarinos et al. (2016), they show that the third player receiving the first pass or second pass after the screen takes 28 and 7% of the shots, respectively. The tactic also explains that a pass to the perimeter (35%) results in a shot more often than a pass to the screener (22%).

The interviewees state that “it is a fairly correct percentage and that it is quite adjusted to the reality of modern basketball” (coach 5). As logic, they interpret that “the other 49% corresponds to a shot, a 1 × 1, a penetration or a continuation of the two players of the screen,” and, therefore, assumes it as an expected result (Figure 5A, quadrant I, category D2152).

Confirming the results of the polar coordinate conducted on the in-depth interviews shown in Figure 5B (quadrant I, category D211), the coaches also deepen their response thinking about the characteristics of the players who constituted Unicaja’s teams and assume that “we had no players playing above the hoop to throw the ball up. and we had no creative point guards on the first pass” (coach 4). However, on the other hand, technicians claim that they had “advantage to play after screening” (coach 4), and that option is reflected in our study by the number of screens where a second pass and a third pass have been used.

*Question 11: In  Unicaja  (10/11),  *pick and repick*  (re-screen) were  observed  in  53  records  (5%  of  the total pick-and-rolls). What do you think of this number of *repicks*? Why?*

The screeners’ actions after the screen are related to less effectiveness when doing a re-screen (*repick*). This tactical behavior is used to create more space and a new possibility for the dribbler, but it generally occurs when the defender follows the dribbler, constraining offensive options. Conversely, the screeners’ following action after the screen with higher effectiveness is continuing to the basket (Gómez-Ruano et al., 2015).

The study by Polykratis et al. (2010) concluded that 3% of screen actions registered were carried out with the movement *pick and repick*; it is a lower value if we compare it with the 7.8% registered in our analysis.

Regarding the option of carrying out this technical-tactical action, most of the interviewees think that it is a scarce result, but in their justifications, it is possible to interpret the reason for that low percentage of re-screen.

It is observed that the coaches have sought their main arguments in the characteristics of the team and the way of playing implemented in the Malaga team to explain the data obtained, which is also reflected in the results of the polar coordinate carried out in the interviews (Figure 6A, quadrant I, category D2131; quadrant III, category D211).

The coaches assume that “the Unicaja players who performed screens were very good shooters, and then the defenses almost never came behind, so the re-screen option was not necessarily usable” (coach 5).

*Question 12: In Unicaja (10/11), 15% of the pick-and-roll was used  with  the  intervention  of  a second screener (used or not). *Horns’*  tactical  work  has  been  a  classic in modern basketball. Would you expect another result? Why?*

Coaches, at all levels, having full responsibility for the performance of their teams, confront a number of challenges regarding the designation of the type of offense they should use during a season of competition. One of the main challenges for researchers and coaches is the detection and prediction of effective team cooperation or individual behaviors, aiming at a more effective execution against the opponent (Garganta, 2009).

However, it is a higher result when compared with the 5% of *double high* recorded in the study by Polykratis et al. (2010).

The tactical work of *horns* has been a classic in modern basketball, and to better understand these percentages, some of the comments expressed in the interviews with experts regarding question 12 are shown below.

For the elite coaches, it is a tactical option that allows two screeners to be positioned so that later “they can attack to one side or the other and choose to play with the power forward or with the center” (coach 4). The polar coordinate of the interviews indicates that the categories D211 and D232 have statistical significance showing that for the coaches, the type of squad they had (Figure 7, quadrant II, category D211) and the space to execute it are essential factors to effectively carry out the action of the double screen (Figure 7, quadrant I, category D232).

Perception, prediction, and execution are essential features of equal value in offensive cooperation (Knight and Newell, 1989; Roca et al., 2011; Gorman et al., 2013; Van Maarseveen et al., 2018).

*Question 13: In  Unicaja  (10/11),  we  observed that the team performed  more  pick-and-roll  actions  in  possessions   where they  were  winning  the  match  (524 possessions)  compared to possessions  where  the team was losing (506 possessions). Is the strategy  of  using pick-and-roll conditioned by the result on the scoreboard?  Is it a deliberate resource, or is it a random piece of information?*

In the last decade, game-related statistics and the notational analysis method were highly considered to define the profile in basketball and their consequent practical utility (Gómez-Ruano et al., 2006, 2016; Ibáñez et al., 2008). Winning or losing in a basketball game is usually tried to be explained by team statistics (Çene, 2018).

The score-line variable showed no significant relationships with ball screen effectiveness. In score-line, the most effective score was −10 to 0, so basically, when one team is losing, they have more effectiveness than if they are winning (between 0 and 10 points). One of the reasons could be that when a team is losing, usually they focus on the way they perform the ball screen and the way they play that is more aggressive to score and generate disorder (defensive unbalance) (Vaquera et al., 2016). These results enhance the importance of players’ interactions during a basketball ball possession that may influence the strategic and tactical approaches to score, particularly team-tactical behaviors, such as screens on the ball (Remmert, 2003).

Gómez-Ruano et al. (2010) suggest the importance of group offensive tactics with the performance of screens and multiple screens to win matches, and the study carried out by Koutsouridis et al. (2018) shows that pick-and-roll can affect the outcome of a game (*p* = 0.001).

The pair of coaches 1 and 5 was quite surprised with the result obtained, and both hoped that more screens were made when they won a game and affirmed that “it is not a random data, because the fact of using more screens when you are winning than when you are losing has some tactical and technical sense,” which confirms and reinforces the opinion of the other experts through the polar coordinate shown in Figure 8A (quadrant II, category D281). In addition, coach 1 explains that the use or not of the pick-and-roll “depends on which is the action that transmits the most confidence to the coach.”

The technical duo of coaches 3 and 4 also showed their surprise on the result obtained but mainly because of the difference between the number of screens when winning or losing a game. Coach 3 affirms that the pick-and-roll “is not something that is used more if you are winning. It is a resource that is used whether you are winning or losing,” and that is why he thought that this fact is more of an effect of a chance than caused or thought out, but the polar coordinate confirmed this (Figure 8B, quadrant IV, category D291). The head coach also did not believe that the use of screening “is a question of the coach’s confidence” (Figure 8B, quadrant IV, category D2143) but is more of an anxiety issue about wanting to control the game because “the pick-and-roll is part of the resources so you take advantage,” and, thus, control the scoreboard. However, he also argued that coaches try to “attack faster when you are behind the scoreboard, look for faster actions and therefore look for fewer pick-and-roll actions.”

Coach 2, from a coach’s point of view, explains that “it can be totally indifferent that you play more ball screens winning or losing, what I do think is that, if you are losing, especially in key moments, and you need to recover the point difference quickly, I think you have to play the ball screen faster” (Figure 8B, quadrant IV, categories D221 and D251).

*Question 14: In  Unicaja  (10/11),  2-on-1 traps were made in 8% (*n* = 95) of the observed defenses, and a defensive change was made  in  19%  (*n* = 216) . Do you think they are good defensive options? Why?*

Ball screens allow for reacting successfully against different defensive strategies, such as switching and hatching (Polykratis et al., 2010; Gómez-Ruano et al., 2015; Remmert and Chau, 2019).

The number of mismatch situations that occur in a match is high enough to be taken into account and prepared in training. When an offensive team encounters a situation of a defensive switch with a mismatch, the shorter the duration, the more beneficial to them. This situation, known as a mismatch, can be a good defensive option to force the offense to play differently, giving rise to different scenarios (Lorenzo et al., 2017).

Finally, in the pick-and-roll now, in spite of the fact that it appears to be the most common offense, some help may be given to various defensive players who are near the key, which will prevent the ball handler from increasing his success percent (Stavropoulos and Stavropoulos, 2020).

In 1,588 (71%) observations, no direct mutual aid action was recorded between the defensive interveners after the ball screen (Nunes, 2020). Santana (2016) agrees with the result of our study, but admits that switch defense is also a valid option to minimize the consequences that an attack seeks with this technical-tactical action.

For the option of switch defense, Coello (2005) and Ivanovic (2006) suggest that it will be used in final possession situations or when the screener and the ball handler are good shooters. Polykratis et al. (2010) present in their work a result of 39.7% of actions with switch defense on ball screens and argue that when a change is made, important defensive imbalances occur.

In the interviews with both coaches 3 and 4, we observed that the team had a well-defined strategy to use the 2-on-1 trap and switch defense options, referring to characteristic defensive technical and tactical concepts used by their players (Figure 9B, quadrant IV, categories D262 and D272).

For coach 2, “ball screen is such an important resource in the attack that it has to have different ways of defending it.” That is why the experts assume that it is also important to develop a defensive technique for the pick-and-roll, and this is reflected in their responses that are observed and shown in Figure 9A (quadrant II, category D262) from the polar coordinate of the interviews. “First because it gives you variety, and second because the two options facilitate doing something different in the defense of the ball screen.”

The polar coordinate analysis (Figure 9B, quadrant I, category D222) also confirms that for the coaches, the exact time of performing these types of actions is essential to achieve defensive success.

In short, the coaches confirm that these are valid defensive resources or variants, but with very unique characteristics; for a 2-on-1 trap to inhibit a specific offensive player or a certain position on the field, the switch defense must be executed by dynamic players and in line with the remaining time of possession.

*Question 15: In Unicaja (10/11), the main defensive responses of the ball handler’s defenders were *chase* (38%; *n* = 439), *over the top* (23%; *n* = 261), and *under the screen* (18%; *n* = 205). For you, what are the best options to defend the pick-and-roll? Why?*

The difference between a good ball handler and a very good player is the ability of the latter to create by himself the best conditions for a shot or penetration toward the basket, as well as to create favorable offensive situations for his teammates (Hill, 1999). A defensive mismatch situation can be created when the ball handler accepts a correct pick from his teammate and penetrates from the right or left side toward the basket (Lorenzo et al., 2017). After a ball screen action, the ball handler has several offensive options; however, the defense also has its tools to inhibit rival effectiveness.

Gómez-Ruano et al. (2015) highlight in their study that the action influence of the ball handler’s defender has increased defensive efficiency when he has fanning/denying the screen, and has decreased when this defender passes from 3rd (under the screen) or 4th (squeeze).

Battaglia et al. (2009) have used an individual defensive nomenclature similar to that of our research and have come to the conclusion that, in most of the screens, the ball handler’s defender chases his offensive player, however, it is more efficient when he passes it from 4th. These authors state that when observing the significance of the results, it is found that only the action of going beyond 2nd is really significant.

Question 15 affects the main defensive responses of the defender of the ball handler. In elite basketball, we try to minimize mistakes and offer few offensive opportunities to the opposing team. In this question, the head coach (coach 3) interprets that the basis for making a good decision is found while both “scouting and at the time of the match.”

Basketball experts agree that a defensive aid strategy depends directly on the time and mainly on the space of the action, in which the polar coordinate corroborates, and that it can condition certain ways of defending the ball handler (Figure 10, quadrant II, category D232).

Synthesizing the answers of the experts, they prefer “option to chase, because the ball screen is already the action that generates the most advantage, since any type of player can play it, but it will be played more by the player who can have the bigger threat of shooting” (coach 5). In elite basketball, they try to minimize errors and offer the least number of offensive opportunities to the rival team, and for the technician, the options of going third (under the screen) or fourth (squeeze) will lower their percentage “because the further you go from the ball, the theoretically easier it is for the offense player.” However, this choice does not inhibit all offensive options of the pick-and-roll, since “going overhead (over the top) obviously removes the shot option but favors the penetration option” (coach 6).

*Question 16: In Unicaja (10/11), the main defensive responses of  the  screener’  defender were *open* (41%; *n* = 466), *show* (36%; *n* = 411), and *hedge* (15%; *n* = 176). For you, what are the best options to defend the direct pick-and-roll? Why?*

Regarding main defensive responses of the screener defender, it is interesting to observe how elite coaches look for the best options for their teams, adapting to the characteristics of their roster, style of play in the league, their rivals, and popular tendencies of the moment (Figure 11B, quadrant I, categories D2141 and D2142; quadrant III, category D2144). In the interview of coach 3, we were surprised to hear that “I don’t like the horizontal *flash* (show) now, as much as it was done before and note that it has been the one I used the most! Right now I wouldn’t do it because I think it’s a defense where you go too far to almost make a triangle when it comes to recovery. I like more to do the vertical *flash* (hedge), stop the ball and recover in the same line.”

Action in pick-and-roll is very fast, and for this reason, synchronization of the moves of offensive players is very important, so that defensive players are not given enough time to react (Stavropoulos and Stavropoulos, 2020). For this reason, coach 2 defends that the best option to combat the offensive effectiveness of the pick-and-roll “is to have different options” (Figure 11A, quadrant I, category D282), and that the other key would be “the screener defender has to be as close as possible to the screen at the moment of blocking” (Figure 11A, quadrant I, category D232). That is why he advocates for defense in *flash*. In addition, statistics is an instrument widely used by basketball technicians, and, in this question, it has been no exception to argue their thoughts through statistical data, a fact confirmed by the polar coordinate in Figure 11A (quadrant I, category D2152).

Furthermore, the most used type is the head-on screen; however, defense is very effective in this case. Screeners’ defenders often use a hedge to defend the screen, and if we look for a relationship with frequency, we can conclude that hedge, show, and open are the most effective ways to defend it, as supported by the study of Remmert (2003). This type of defense is characterized by great aggressiveness and concern of the ball handler to penetrate.

In line with this perspective, the study by Battaglia et al. (2009) also coincides with the study by Refoyo et al. (2007), in which the short defensive flash appears strongly associated with defensive success.

Manzano et al. (2005) conclude that when the pick-and-roll occurs, there are alterations in the structure of both teams. The match observation suggests that the pick-and-roll is used to decrease the defense pressure regarding carrying of the ball, offensive transition, and development of a quick attack, organized attacks at the beginning, besides defensive responses that each team implements in every action.

However, this study is not exempted from limitations since it is based on data from the 2010–2011 season. This is justified by the fact that it is difficult for coaches to publicly reveal their tactical approaches or those of their teams for ethical and strategic reasons. Over time, this information maintains its scientific significance and reduces its impact in the field of sports.

Finally, looking toward the future, it would be interesting to consider if the speed and rhythm of execution of ball screens depend on the result of the scoreboard by T-pattern analysis, as has been conducted on other sports (Pic and Jonsson, 2021).

## Conclusion

This study shows that the application of mixed methods, by polar coordinate analysis of the coding made to the responses on a systematized interview, is an effective strategy for obtaining relevant information on the expert knowledge of elite coaches on the influence of the pick-and-roll in tactical actions in basketball. It is innovative, from the perspective of mixed methods. In the past, the answers of interviews were analyzed qualitatively, but here we have analyzed the data quantitatively.

The interview confirms that coach 1 and his staff were less confident than coach 3 in the options that pick-and-roll offers, which is also reflected in the record of screens made and simulated. Coaches consider ball screens as an advantage-creating action to read the opponent and end or maintain the preset game action. This is why screens increase their frequency at the start of offensive systems. Coaches rely on ball screens as a last resort when possession time is short to seek an immediate shot option. The experts conclude that controlling the ball with the dominant hand facilitates the execution of the pick-and-roll, but that it is not a decisive or limiting aspect.

Seventy-three percent of the team’s offensive actions after a timeout were performed through a pick-and-roll. The elite coaches assume that a timeout offers the necessary time to think about the previously prepared baseline or sideline play and resort to head-on screen preferring to build a conservative, solid offensive situation that allows for success. In moments of tension and with an unfavorable or even result on the scoreboard, offensive systems become more important and coaches rely on ball screens to try to control the dynamics of the game.

We highlight in the defensive section that the coaches confirm the defense switch action and the 2-on-1 trap as valid defensive resources or variants but have very unique characteristics; a 2-on-1 trap inhibits a specific offensive player or a certain position on the field; defense switch must be executed by dynamic players and in line with the remaining time of possession. The elite coaches conclude that the defensive response of the defender of the ball handler depends on the characteristics of the offensive player, moment of the game, distance to the basket, and previous scouting. They corroborate their preference for the option to chase, because it is the most aggressive action. For the defensive response of the screener defender, high-level coaches prefer the *flash*, mainly the vertical one (hedge), to provoke an error and take the initiative in defense. This decision will be defined by the agility and aggressiveness characteristics of the defender in question (screener).

## Data Availability Statement

The raw data supporting the conclusions of this article will be made available by the authors, without undue reservation.

## Ethics Statement

The studies involving human participants were reviewed and approved by Ethics Committee for Clinical Investigations of the Sports Administration of Catalonia (24/20118/CEICEGC). The patients/participants provided their written informed consent to participate in this study. Written informed consent was obtained from the individual(s) for the publication of any potentially identifiable images or data included in this article.

## Author Contributions

HN documented, designed, drafted, and wrote the manuscript. LD documented and drafted the manuscript. XI and MTA supervised the method and procedure sessions and statistical analyses, and revised the manuscript for theoretical and intellectual content. Finally, all authors provided the final approval of the version to be published.

## Conflict of Interest

The authors declare that the research was conducted in the absence of any commercial or financial relationships that could be construed as a potential conflict of interest.

## Publisher’s Note

All claims expressed in this article are solely those of the authors and do not necessarily represent those of their affiliated organizations, or those of the publisher, the editors and the reviewers. Any product that may be evaluated in this article, or claim that may be made by its manufacturer, is not guaranteed or endorsed by the publisher.
